# Bloom announcement: Late season cyanobacterial blooms co-dominated by *Microcystis flos-aquae, Lyngbya birgei*, and *Aphanizomenon flos-aquae* complex in Hamilton Harbour (Lake Ontario), an area of concern impacted by industrial effluent and residential wastewater.

**DOI:** 10.1016/j.dib.2021.106800

**Published:** 2021-01-27

**Authors:** Arthur Zastepa, Camille Chemali

**Affiliations:** Environment and Climate Change Canada, Canada Centre for Inland Waters, Burlington, Ontario, Canada

**Keywords:** Cyanobacterial and harmful algal bloom, Microcystins, Anatoxins, Lake Ontario, Hamilton Harbour, Area of concern

## Specifications Table

**Table:** 

Subject	Aquatic Science
Type of data	Table Image Chart Graph Figure
How data were acquired	Phytoplankton community composition was obtained with an inverted microscope using a modified Utermöhl method [14]. ELISA-based cyanobacterial toxin analysis was done using microcystins-ADDA polyclonal antibody – enhanced ELISA kit (Product No. 520011, Abraxis, Warminster, PA, USA) and anatoxin-a ELISA Microtiter Plate Kit (Product No. 520060, Abraxis). Cyanobacterial toxin analysis was also done by UPLC-MS/MS (Xevo G2-XS QTof MS, Waters Corp., Milford, MA, USA).
Data source location	City/Town/Region: Ontario Country: Canada Name of Body of Water: Hamilton Harbour in Lake Ontario Latitude and longitude (and GPS coordinates) for collected samples/data: Station Bayfront Park Beach (BF): 43.271573; -79.874499
Data format	Raw Analyzed
Identification	Whole water samples were preserved with Lugol's iodine for identification and enumeration with an inverted microscope using a modified Utermöhl method [14].
Strain Characteristics	N/A
Data accessibility	Repository name: Mendeley Data Data ID / Accession number: http://dx.doi.org/10.17632/nytpb5mrfm.1 Direct URL to data: http://dx.doi.org/10.17632/nytpb5mrfm.1
*Compositional Profile of the Strain's Biomass*	
Lipid Profile	Not available
CHNO Analysis (if available)	Not available
Protein, Carbohydrate, Lipid, Ash Content (if available)	Not available
Protein and Amino Acid Profile (if available)	Not available
Carbohydrate Profile (if available)	Not available
Toxin Concentrations (*µ*g/L) (if available)	Total Microcystin Concentration Station Bayfront Park Beach (BF): July 19, 2017 < 40 ng/L August 9, 2017 < 300 ng/L September 14, 2019 < 36,000 ng/L October 18, 2017 < 70 ng/L November 15, 2017 < 20 ng/L Anatoxin-a Concentration: July 19, 2017 < 200 ng/L ELISA method detection limit (MDL) was 0.1 ng/L for total microcystins (based on microcystin-LR). UPLC-MS/MS MDL was between 0.1 and 1.5 ng/L for microcystins, depending on variant and 200 ng/L for anatoxin-a.
References to Methods Used for Profiling	Not available

## 1 Introduction

Hamilton Harbour is a large embayment (approximately 22 km^2^, over 2.8 × 10^8^ m^3^, maximum depth 23 m, mean depth 13 m) at the western part of Lake Ontario with a long history of anthropogenic impacts, particularly from industrial effluent and residential wastewater [1]. Designated an area of concern (AOC) under the Great Lakes Water Quality Agreement (GLWQA) in 1987, it continues to suffer from the buildup of hazardous chemicals in the water and sediments as well as eutrophication [3], [4], [5]. Several creeks (Spencer Creek, Redhill Creek, Grindstone Creek, Chedoke Creek) contribute to total water inflow (∼9.7 m^3^/sec) but a significant volume originates from two large wastewater treatment plants - the Woodward Avenue Wastewater Treatment Plant (∼40%) servicing the city of Hamilton and the Skyward Wastewater Treatment Plant servicing the city of Burlington (∼13%) [6]. The most significant water exchange between Hamilton Harbour and the rest of Lake Ontario is via a narrow channel on the east side of Hamilton Harbour, the Burlington Shipping Canal [1].

**Fig. 1:**
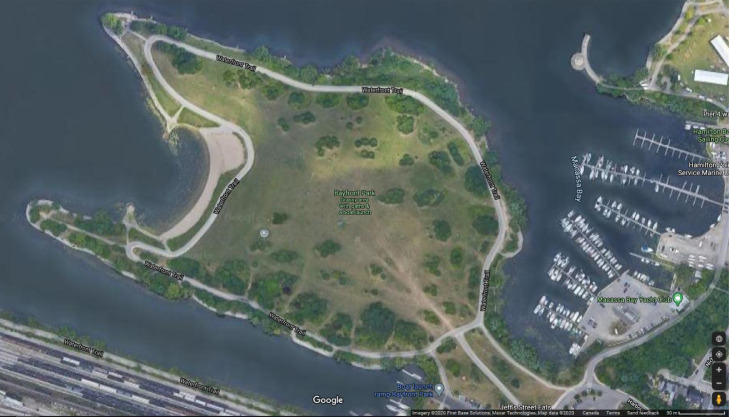
Satellite imagery (aerial view) of Bayfront Park Beach, Hamilton Harbour, Lake Ontario (Ontario, Canada) where samples of the 2017 cyanobacterial and harmful algal blooms were collected. Note the crescent shape of Bayfront Park Beach and adjacent parkland, making it susceptible to localized accumulations of material.

**Fig. 2:**
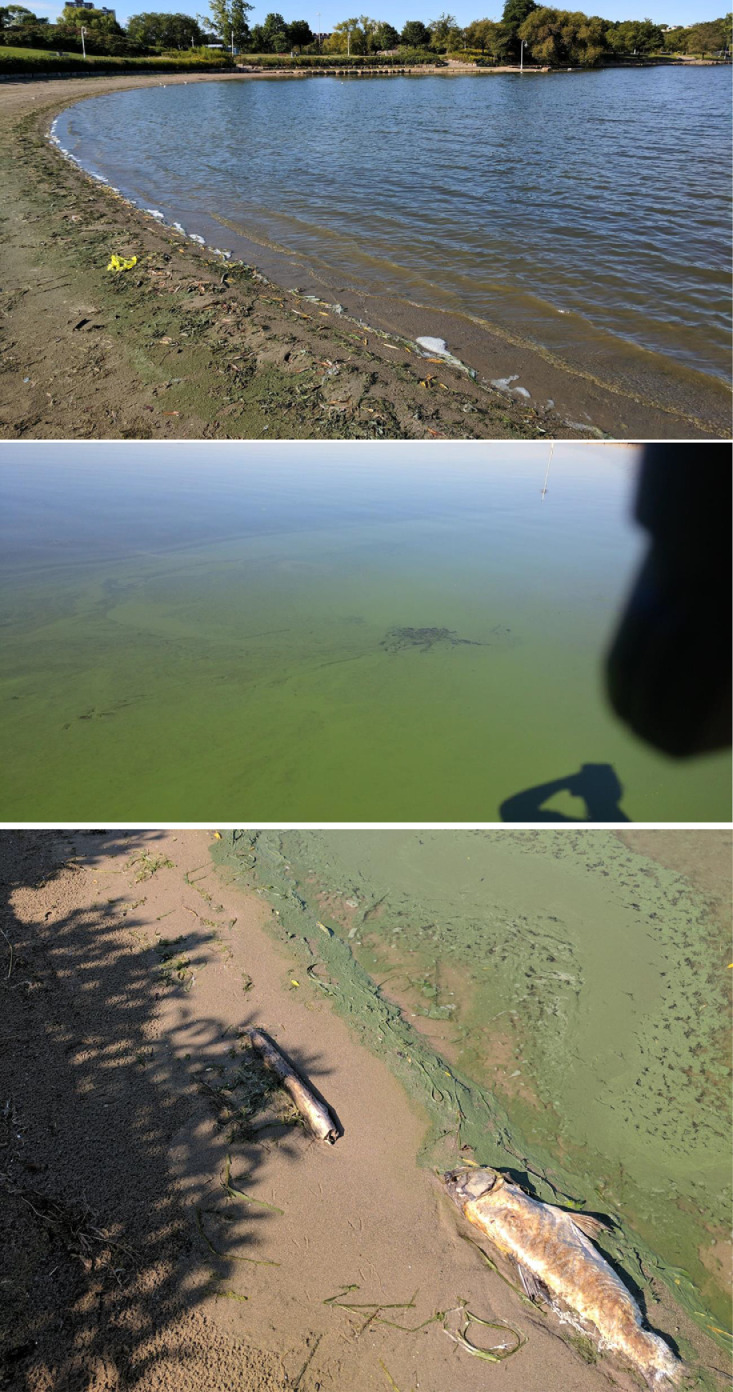
Photographic documentation of the cyanobacterial and harmful algal blooms at Bayfront Park Beach in Hamilton Harbour, Lake Ontario (Ontario, Canada) (September 2017). Top panel highlights the crescent-shaped shoreline susceptible to accumulation of phytoplankton biomass on the sand. Middle panel shows the visual characteristics (e.g. discolouration, streaking, heterogeneity) of the cyanobacterial bloom in the water along the shoreline of Bayfront Park Beach. Bottom panel documents one of several sightings of dead fish along the cyanobacterial bloom at Bayfront Park Beach.

**Fig. 3:**
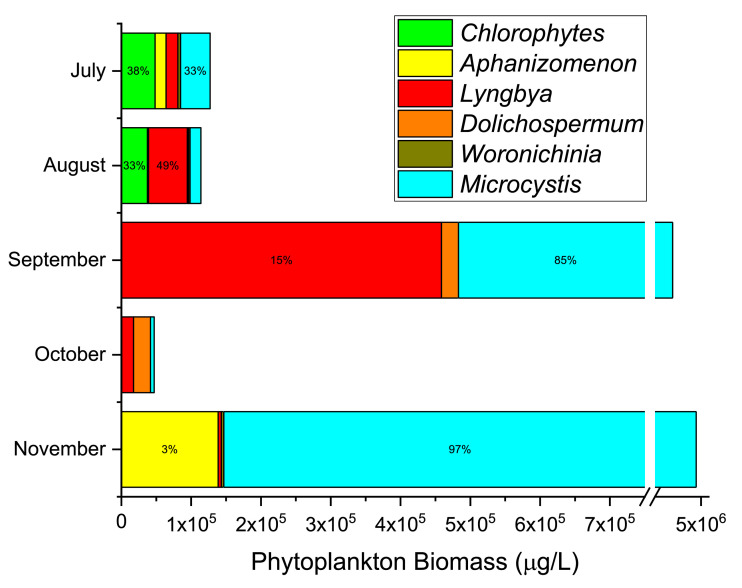
Total biomass (μg/L), with relative proportions (%), of major taxonomic groups within water samples collected from Bayfront Park Beach, Hamilton Harbour, Lake Ontario (Ontario, Canada) from July to November 2017. The dominant group, cyanobacteria, are identified to the level of genus. Based on data upload to Mendeley Data repository [2].

In 2017, cyanobacterial and harmful algal blooms (cHABs) were observed and characterized along Bayfront Park Beach in Hamilton Harbour, Ontario, Canada (Fig. 1, Fig. 2). The beneficial use of Bayfront Park Beach has been classified as impaired for beneficial use impairment (BUI) #8, *Eutrophication or Undesirable Algae*, and BUI #10, *Beach Closings and Water Contact Sports*, under the Hamilton Harbour's Remedial Action Plan. As part of Environment and Climate Change Canada's Great Lakes Action Plan, monthly sampling of whole water grabs, within and outside the blooms, occurred from July to November inclusive. In July and August a mixed community of chlorophytes and cyanophytes dominated the bloom, which exceeded 100 mg/L in phytoplankton total biomass (TB) (Fig. 3) [2]. By September, this already high TB increased dramatically beyond 3,000 mg/L before subsiding below 50 mg/L in October, a likely consequence of more turbulent waters in early autumn (Fig. 3). The community in September was dominated by *Microcystis* (60% TB *M. flos-aquae* and 16% TB *M. aeruginosa*) and *Lyngbya* (15% TB *L. birgei*). In November, TB exceeded 4,500 mg/L, with *M. flos-aquae* expanding its dominance (95% TB) while *L. birgei* was replaced by *Aphanizomenon flos-aquae*, albeit with reduced biomass (3% TB) (Fig. 3). It is suspected, based on spatiotemporal observations of bloom dynamics and circulation patterns within Hamilton Harbour, that phytoplankton biomass proliferates offshore and is transported by winds and currents to Bayfront Park Beach, where it is captured and accumulates within its crescent-shaped shoreline (Fig. 1, Fig. 2) [7].

## 2 Environmental Impact

• These recurrent cHABs are thought to contribute to the development and persistence of hypoxia/anoxia in the hypolimnion of Hamilton Harbour, which could negatively impact the habitat of residing cold-water fish [8].
• Fish deaths are regularly observed along Bayfront Park Beach and other areas of Hamilton Harbour (Fig. 2, bottom panel), especially from August to October, and can occur as a consequence of the cyanobacterial toxin release, anoxia, and pathogen growth (e.g. *Clostridium botulinum*) associated with decaying bloom material.

## 3 Toxicity Information

Microcystins concentrations exceeding recreational guideline (>20,000 ng/L) were observed, with LR, LA, and RR the dominant variants [9]. Remarkably, microcystins were detected into November, highlighting their persistence. Anatoxin-a was detected in July only. Taxa present are known to produce other bioactive metabolites that can affect ecosystem health and additional screening is recommended [9].

## 4 Economic Impact

• In 2016, Bayfront Park Beach was closed to swimming under the direction of the City of Hamilton Public Health Services Department due to a history of poor water quality, citing recurrence of microcystin-producing cyanobacteria [10].
• In subsequent years, accumulations of phytoplankton bloom biomass and associated odours have driven the City of Hamilton to use vacuum trucks to clean up the shorelines around marinas, beaches, and other recreational locations (e.g. Royal Botanical Gardens, Cootes Paradise, Macassa Bay Yacht Club) [11]. This material has been released back into sanitary sewers, putting additional pressure on the Woodward Avenue wastewater treatment plant.

## 5 Experimental Design, Materials and Methods

Sampling within Bayfront Park Beach, Hamilton Harbour (Ontario, Canada) occurred from July to November, inclusive, and samples were collected just below the surface at a 0.5 m depth using a 4 L HDPE bottle. Concurrently, physicochemical parameters (pH, conductivity, temperature, dissolved oxygen) were measured using a 600QS sonde (YSI Inc., Yellow Springs, OH, USA). Sampling was completed in the morning between 9:30 h to 12:00 h.

Whole water samples were concentrated onto GF/C filters (1.2 µm pore size, 47 mm diameter) and stored at −80 °C in the dark until extraction. Extraction of cyanobacterial toxins was done in 10 mL of analytical grade aqueous methanol (1:1 v/v) amended with analytical grade formic acid (0.1%) with the use of probe sonication (30 s on, 30 s off, repeated three times) (Fisher Scientific Co. Qsonica Sonicator Q500, Ontario, Canada). Samples were then centrifuged at 3000 rpm for a total of 15 min to pellet debris. A PTFE syringe filter (1.0 µm pore size, 30 mm diameter) attached to a 10 mL gas-tight glass syringe was used to filter the resulting supernatant into a glass vial, which was then evaporated to dryness using nitrogen gas-flow and heat (30 °C). Resulting residue was reconstituted with 1 mL of analytical grade aqueous methanol (1:1 v/v) and vortexed. A final filtration was done using a PTFE syringe filter (0.45 µm pore size, 15 mm diameter) attached to a gas-tight glass syringe into a 1.5 mL HPLC amber glass vial and stored at −80 °C in the dark until analysis.

Enzyme-linked Immunosorbent Assay (ELISA) was used for determination of total microcystins and anatoxins, according to manufacturer's instructions (PN 520011 and PN 52255B, respectively, Abraxis Inc., Warminster, PA, USA). Briefly, a 96-well microtiter plate was used to conduct analysis in triplicate, using 50 µL of sample or standard each time. A Synergy HTX multi-mode reader with Gen5™ software (Bio-Tek, VT, USA) was used to measure the absorbance at 450 nm [12].

High performance liquid chromatography (Agilent Technologies, ON, Canada) coupled with mass spectrometry (AB Sciex, USA) was used to identify most abundant microcystin variants and confirm presence of anatoxin-a using previously optimized conditions and parameters [13]. Briefly, reverse-phase chromatography (C-18) was performed with an elution gradient of mobile phase (90% → 10% acetonitrile amended with formic acid and ammonium formate at 0.3 mL/min with similarly amended water as polar component) to separate analytes prior to electrospray ionization in positive mode. A triple quadrupole arrangement and multiple reaction monitoring (MRM) was used to generate and monitor two precursor to product transitions for each microcystin variant [13]. Quantitation was done by Analyst® software and based on area under the peak using external calibration curves generated from nine microcystin variant standards including microcystin LR, 7dmLR, LA, WR, LY, LF, YR, LW, and RR.

Lugol's iodine solution (2% v/v) was used to preserve aliquots of 100 mL unfiltered sample water for taxonomic identification and enumeration. Taxonomic identification and enumeration of abundance, biomass, and biovolume were done using the Utermöhl technique as described previously [14].

## 6 Funding Information

This study was supported by Environment and Climate Change Canada's Great Lakes Action Plan and the Hamilton Harbour Remedial Action Plan.

## CRediT Author Statement

**Arthur Zastepa:** Conceptualization, Project administration, Supervision, Investigation, Data curation, Writing – Original draft preparation, review, and editing, Funding acquisition; **Camille Chemali:** Data curation, Writing – review and editing.

## Declaration of Competing Interest

The authors declare that they have no known competing financial interests or personal relationships which have, or could be perceived to have, influenced the work reported in this article.

## Appendix Supplementary materials

Image, application 1

Image, application 2

Image, application 3

## References

[1] Barica J. (1989). Unique Limnological phenomena affecting water quality of Hamilton harbour, Lake Ontario. J. Great Lakes Res..

[2] Zastepa, Arthur; Chemali, Camille. Bloom announcement: 2017 Hamilton Harbour Lake Ontario (Ontario Canada) Cyanobacterial Bloom, Mendeley Data, v1 (2020) doi:10.17632/nytpb5mrfm.1. PMC7868921 33598512

[3] Rao Y.R., Marvin C.H., Zhao J. (2009). Application of a numerical model for circulation, temperature and pollutant distribution in Hamilton Harbour. J. Great Lakes Res..

[4] Hiriart-Baer V.P., Milne J., Charlton M.N. (2009). Water quality trends in Hamilton Harbour: two decades of change in nutrients and chlorophyll a. J. Great Lakes Res..

[5] Hall J.D., O'Connor K.M. (2016). Hamilton harbor remedial action plan process: connecting science to management decisions. Aqu. Eco. Hea. Man..

[6] Gudimov A., Stremilov S., Ramin M., Arhonditsis G.B. (2010). Eutrophication risk assessment in Hamilton Harbour: system analysis and evaluation of nutrient loading scenarios. J. Great Lakes Res..

[7] Yerubandi R.R., Boegman L., Bolkhari H., Hiriart-Baer V. (2016). Physical processes affecting water quality in Hamilton Harbour. Aqu. Eco. Hea. Man..

[8] Pothoven S.A., Vanderploeg H.A., Höök T.O., Ludsin S.A. (2012). Hypoxia modifies planktivore-zooplankton interactions in Lake Erie. Can. J. Fish. Aquat. Sci..

[9] Canada Health (2012). Guidelines for Canadian recreational water quality.

[10] City of Hamilton. 2019. Beach Monitoring Report prepared by Hamilton Public Health Services. Accessed at https://www.hamilton.ca/parks-recreation/parks-trails-and-beaches/beach-water-quality-in-hamilton

[11] The Hamilton Spectator. 2018. City of Hamilton still sucking sewage in Chedoke Creek. https://www.insidehalton.com/news-story/8758454-city-of-hamilton-still-sucking-sewage-in-chedoke-creek/

[12] Ficher W.J., Garthwaite I., Miles C.O., Ross K.M., Aggen J.B., Chamberlin A.R., Towers N.A., Dietrich D.R. (2001). Congener-Independent immunoassay for microcystins and nodularins. Environ. Sci. Technol..

[13] Zastepa A., Pick F.R., Blais J.M., Saleem A. (2015). Analysis of intracellular and extracellular microcystin variants in sediments and pore waters by accelerated solvent extraction and high performance liquid chromatography-tandem mass spectrometry. Anal. Chim. Acta.

[14] Findlay D.L., Paterson J.J., Hendzel LL L.L., Kling H.J. (2005). Factors influencing Gonyostomum semen blooms in a small boreal reservoir lake. Hydrobiologia.

